# Repeat hepatic resection combined with intraoperative radiofrequency ablation versus repeat hepatic resection alone for recurrent and multiple hepatocellular carcinoma patients meeting the Barcelona Clinic Liver Cancer stage A: A propensity score‐matched analysis

**DOI:** 10.1002/cam4.5662

**Published:** 2023-02-01

**Authors:** Yang Huang, Liangliang Xu, Min Huang, Li Jiang, Mingqing Xu

**Affiliations:** ^1^ Department of Liver Surgery, Liver Transplantation Center West China Hospital of Sichuan University Chengdu China

**Keywords:** hepatic resection, multifocal tumors, radiofrequency ablation, recurrent hepatocellular carcinoma, survival outcome

## Abstract

**Background:**

The surgical indications and therapeutic strategies for early‐stage multifocal and recurrent hepatocellular carcinoma (rHCC) remain controversial. This study compared the long‐term outcome of patients with recurrent and multifocal HCC meeting the Barcelona Clinic Liver Cancer (BCLC) stage A with repeat hepatectomy (RH) and RH combined with intraoperative radiofrequency ablation (RFA).

**Methods:**

A total of 109 consecutive patients with intrahepatic early‐stage multifocal rHCC within BCLC stage A following RH or RH + RFA were retrospectively collected from April 2010 to May 2020. Propensity score matching, subgroup analysis, and univariate and multivariate analyses were performed. Overall survival after recurrence (rOS) and recurrence‐free survival after recurrence (rRFS) were calculated by Kaplan–Meier analysis.

**Results:**

The 1‐, 3‐, and 5‐year rOS and rRFS of the combination group and the RH group were similar (*p* = .699; *p* = .587, respectively). The similar results also appeared in matched population. Subgroup analyses indicated that there was no significant difference between patients with two tumors and three tumors, but the RH group was associated with better rRFS than the combination group for patients whose tumors were located in the same lobe (*p* = .045). Multivariate analysis revealed that time to recurrence (TTR) ≤ 2 years and intrahepatic metastasis (IM) pathologically were independent risk factors.

**Conclusions:**

For multifocal rHCC patients meeting the BCLC stage A, tumor which is difficult to be surgically resected could be treated by RFA in order to avoid complications or bleeding. Tumors which were located in the same lobe may be more suitable to be treated by RH alone.

## Graphical abstract

This figure represents our research of comparing the survival outcomes for multifocal recurrent hepatocellular carcinoma patients meeting the Barcelona Clinic Liver Cancer stage A who underwent repeat hepatectomy alone or repeat hepatectomy combined with intraoperative radiofrequency. The results may propose some new valuable suggestions and treatment decision‐making.

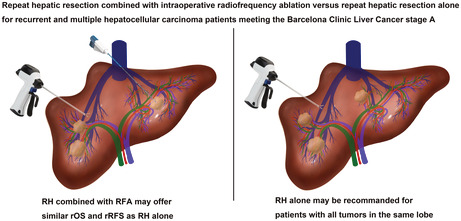

## 1 INTRODUCTION

Hepatocellular carcinoma (HCC) is one of the most common primary malignancies of the liver and is the third leading cause of cancer‐related death worldwide.^1^, ^2^ At present, partial hepatectomy is the recommended first‐line treatment for primary HCC.^3^ Nevertheless, the postoperative tumor recurrence rate is not acceptable; the 5‐year recurrence rate is 60%–80% following primary resection with curative intent, and 80%–95% of recurrences are confined to the remnant liver.^4^, ^5^, ^6^, ^7^ Future treatment for HCC will mainly focus on rHCC. Many studies have reported that 5‐year survival after RH resembles that after initial hepatectomy.^8^, ^9^, ^10^ However, guidelines for the management of early‐stage rHCC remain controversial and poorly defined.

Improvements in recurrence surveillance and medical imaging have led to the diagnosis of rHCC at an early stage when various treatments may still be available. rHCC usually originates from IM or multicentric occurrence (MO).^11^ IM refers to HCC foci developing from tumor cells that have spread into the remnant liver via the portal vein before or during hepatic resection, usually occurring early. MO refers to new HCC foci developing due to the existence of chronic active hepatitis, cirrhosis, or other HCC‐relevant risk factors after resection, appearing later. Many researchers regarded the 2‐year recurrence interval as the critical value of early recurrence (ER) and late recurrence (LR), which indicates that the clinical progression and outcomes of these two types of recurrence are significantly different.^12^, ^13^, ^14^, ^15^

Most early‐stage rHCC patients can benefit from surgical intervention on the premise of good liver function. The therapeutic principles used for rHCC are essentially the same as those used for primary HCC.^16^ In that way, the mainstay treatments of early‐stage rHCC include salvage liver transplantation (SLT), RH, RFA, microwave coagulo‐necrotic (MCN), transarterial chemoembolization (TACE), or systemic treatment using targeted therapy or chemotherapy. SLT is the most beneficial option, but it is of limited use in regions with liver donor shortage and disease progression on waiting lists.^17^ TACE combined with systemic treatment was managed for patients who had poor general performance and liver function, severe cirrhosis, and unresectable conditions. RH, RFA, and MCN are the next‐most efficacious treatment options for early‐stage rHCC.^9^, ^10^, ^18^, ^19^, ^20^, ^21^

Currently, many centers use RH as the first‐line treatment for rHCC and have claimed that it is preferable because of its acceptable survival benefits.^9^, ^22^ Unfortunately, RH can be carried out only in rHCC patients within 13.4%–22.9% because of poor liver function and multiple intrahepatic recurrence.^23^ RFA is now the new first‐line locoregional treatment due to its therapeutic effectiveness, stable safety, and minimal liver damage. Some studies have shown that RFA was as effective as RH in the treatment of small rHCC (≤3 cm in diameter) in terms of long‐term survival outcome.^19^, ^24^ In addition, RFA had advantages over RH in terms of lower mortality and compilation rate.^25^

To the best of our knowledge, most studies focused on early‐stage single rHCC, and there was no report published in the medical literature comparing the efficacy of RH and/or RFA for early‐stage multifocal rHCC. Whether RH combined with RFA could make up for the vacancy that could not be achieved by RH alone in the treatment of early‐stage multifocal rHCC. The aim of this retrospective study was to compare the outcome of patients with multifocal rHCC meeting the BCLC stage A after RH combined with intraoperative RFA or RH alone.

## 2 METHOD

This present study was designed as a retrospective review, approved by the West China Hospital Ethics Committee, and conducted in accordance with the ethical guidelines of the Declaration of Helsinki.

### 2.1 Patients

We reviewed the medical records of patients who underwent RH alone or RH combined with RFA for early‐stage multiple rHCC between April 2010 and May 2020 at the Department of Hepato‐Biliary‐Pancreatic Surgery, West China Hospital, Sichuan University, for inclusion in this study. The following patients were excluded from the study: underwent nonsurgical treatments such as TACE and anti‐tumor drug therapy after initial resection and before the first recurrence; received the above nonsurgical treatments after the first recurrence; single recurrent tumor or not up to the BCLC stage A; combined with other therapies, such as SLT and tyrosine kinase inhibitor administration; extrahepatic metastases, severe liver dysfunction, or significant coagulopathy; lost to follow‐up.

They were divided into two groups according to surgical procedures: the combination group, which consisted of those who underwent open RH combined with RFA, and the RH group, which consisted of those patients who underwent open RH alone. The follow‐up data were updated in January 2021 or until death.

### 2.2 Preoperative Evaluation

Hepatologists, surgeons, and radiologists jointly participated in the preoperative evaluation. The potential for resection was evaluated by ultrasonography, computed tomography (CT), and magnetic resonance imaging (MRI). Liver function was assessed by a combination of Child–Pugh score, liver biochemistry, and indocyanine green test. RH was undertaken in the presence of endurable cardiopulmonary and renal function, Child–Pugh grade A or B that could be returned to Child–Pugh grade A by routine treatment, and a normal indocyanine green test at 15 min. In principle, RH was performed to treat superficial lesions while RFA was for deep lesions. However, RFA was excluded if the tumor involved the main bile duct because of the probability of destruction of the major bile ducts by RFA. The indications for RH alone were the presence of an appropriate residual liver volume evaluated by CT or MRI. We also noted well‐preserved liver function and anatomical location as other necessary conditions for RH. If the patient had no sufficient residual liver volume or well‐preserved liver function, then wedge resection was performed. when ICG‐R15 was 10% or less and all tumors were located in adjacent liver segments, en bloc resection was permitted. All treatments were performed after obtaining written informed consent from the patient.

### 2.3 Treatments

The treatment scheme used for early‐stage multifocal rHCC was essentially the same as that used for primary multifocal HCC with a small modification in liver function requirement. RH was assigned when there was the possibility for the complete removal of all tumors while retaining sufficient remnant liver volume. If ICG‐R15 is less than 10%, anatomical extended hepatectomy is permitted. For lesions ≤3 cm in diameter, RFA was also deemed to be the radical therapeutic method. In addition, the indications for performing intraoperative RFA include insufficient remnant volume, deep location in liver parenchyma, and distant anatomical location between tumors. Otherwise, other factors affecting the treatment choice come from the experience and skills of the surgeon. Significant adhesions and ambiguous anatomical structures increase the difficulty of RH and the uncertainty of RFA.

### 2.4 Surgical procedure

All operations were performed using the open approach, starting with an exploration, separating the adhesions caused by the initial operation thereafter. Intraoperative ultrasound was routinely performed to assess the tumor burden, the remnant liver volume, and the possibility of negative resection margin. Parenchyma transection was carried out using a Cavitron Ultrasonic Surgical Aspiration (CUSA; Valleylab Corporation) or clamping crush. The Pringle maneuver was routinely performed with a 15‐min occlusion and 5‐min reperfusion cycle when necessary. Experienced ultrasound doctors and surgeons should jointly locate the tumor by conventional ultrasound or contrast‐enhanced ultrasonography (CEUS). A Cool‐tip RFA system (Valleylab Corporation) was used for ablation. The numbers of overlapping ablations and ablation points were determined by the number and size of tumors. After completing the RFA procedure, we cauterized the electrode path to avoid bleeding and track seeding of the tumor.

### 2.5 Diagnostic criteria and definitions

The clinical diagnosis of HCC at both its initial and recurrent stages was based on the criteria of the American Association for the Study of Liver Diseases (AASLD).^7^ The diagnosis of rHCC was confirmed by histopathology for resection. Some suspicious lesions were pathologically confirmed by ultrasound‐guided biopsy for RFA. The presence of adhesions was defined when extensive adhesiolysis was necessary during surgery.^22^ Curative resection was defined as complete resection of all macroscopically detectable tumors with histological tumor‐free margins along the parenchymal transection line.

Complete RFA ablation was defined as an area equal to or larger than the ablated tumor without contrast enhancement after the RFA procedure at 1 month.

Moreover, based on Couinaud's segmentation, the right lobe of the liver is composed of segments V, VI, VII, and VIII, while the left lobe of the liver is composed of segments II, III, and IV.^26^

According to the Liver Cancer Study Group of Japan with modifications.^27^ MO was diagnosed when the resected recurrent tumors met one of the following modified criteria: (1) The recurrent tumor consists of well‐differentiated HCC only; (2) The recurrent tumor has precancerous lesions or well‐differentiated HCC around the less differentiated HCC and shows a “nodule‐in‐nodule” form; (3) All components of rHCC show higher differentiation than the primary resected tumors. IM was diagnosed when resected rHCC showed either the same or less differentiation than the initial resected tumor.

### 2.6 Follow‐up

In both groups, enhanced CT was conducted 1 month after the treatment to evaluate whether the tumor was completely controlled. Thereafter, ultrasonography was carried out in the outpatient clinic once every 2 months within the first 2 years and then once every 3 months thereafter, while the CT scan was performed every 6 months. At each follow‐up, blood tests, liver function tests and AFP, as well as MRI or CEUS, if necessary, were carried out. Regardless of the serum HBV deoxyribonucleic acid, all patients with hepatitis‐related HCC in our hospital were consulted by a hepatologist for antiviral therapy. Once the second recurrence was confirmed, patients were treated with third resection, RFA, TACE, sLT, or anti‐tumor drugs according to the patients' physical condition, liver function, and treatment intention.

### 2.7 Statistical analysis

Categorical variables are expressed as numbers and percentages (%), and continuous variables are expressed as medians (range). Univariate analysis was performed to use Student's t test or the Mann–Whitney *U* test, chi‐squared test or Fisher's exact test, as appropriate. rOS was defined as the period from the date of RH or RH + RFA to the date of death or the last follow‐up until May 2021. rRFS was defined as the interval between the date of RH or RH + RFA and the date of second recurrence when medical tests confirmed. The rOS and rRFS rates were measured by using the Kaplan–Meier method and compared by using the log‐rank test. The relative prognostic significance of the variables in predicting survival was assessed by Cox proportional hazards regression analysis.

To minimize confounding bias between RH group and combination group, a 1:1 matching method using propensity score (caliper value .02) was performed by R software (Version 2.12x). All tests for differences were two‐tailed, and *p*‐values were considered statistically significant when the associated probability was less than .05. Statistical analysis was performed using the SPSS software program (version 20).

## 3 RESULTS

### 3.1 Baseline data at recurrence

**FIGURE 1:**
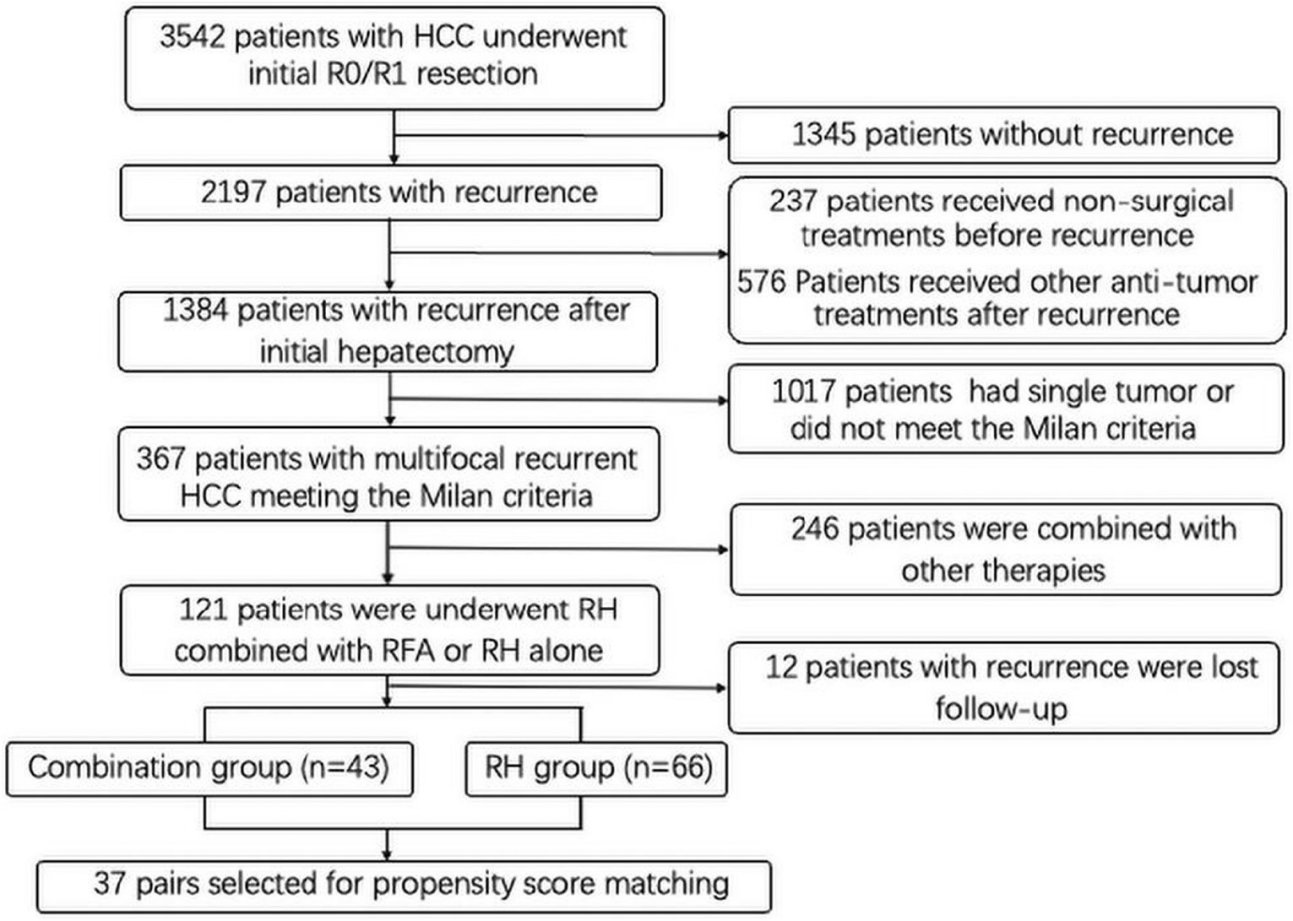
Flow of study participants. HCC, hepatocellular carcinoma, RH, repeat hepatectomy, RFA, radiofrequency ablation, RH, repeat hepatectomy.

A total of 2197 patients with rHCC underwent treatment from April 2010 to May 2020. Finally, a consecutive series of 109 patients conforming to inclusion criteria were enrolled in this study (Figure 1).

The demographic characteristics of the pre‐ and post‐propensity score matching (PSM) cohorts were summarized in Table 1. Before PSM, the number of patients who underwent RH combined with RFA and RH alone was 43 and 66, respectively. Among them, 39 (90.7%) were male, and the mean age was 54.28 years (range, 31–78 years) in the combination group, while 54 (81.8%) were male, and the mean age was 56.23 years (range, 27–85 years) in the RH group. Other indicators, such as hepatitis background, serum AFP level, liver function parameters, prothrombin time, platelet count, total tumor size, and time to recurrence revealed no significant difference between the two groups. More patients in RH group had two tumors than patients in combination group (*p* = .034), the largest tumor size was larger in combination group than in RH group (*p* = .026), and more patients in combination group had tumors located in the different lobes than patients in RH group (*p* = .049). The above parameters revealed no significant difference between the two groups after PSM.

**TABLE 1:** Baseline characteristics of HCC patients with recurrent and multifocal tumors meeting the Milan criteria at the time of recurrence.

Variable	Before propensity matching			After propensity matching		
	Combination (*n* = 43)	RH alone (*n* = 66)	*p* value	Combination (*n* = 37)	RH alone (*n* = 37)	*p* value
Age, years, median (range)	52 (31–78)	56 (27–85)	.400	52 (31–78)	54 (27–79)	.871
Male, *n* (%)	39 (90.7)	54 (81.8)	.200	33 (89.2)	34 (91.9)	1.000
Underlying liver disease						
HBV, *n* (%)	38 (88.4)	56 (84.8)	.602	36 (97.3)	32 (86.5)	.199
HCV, *n* (%)	5 (11.6)	8 (12.1)	.938	1 (2.7)	4 (10.8)	.358
HBV DNA ≤1000, IU/mL, *n* (%)	5 (11.6)	4 (6.1)	.302	4 (10.8)	3 (8.1)	1.000
Serum AFP ≥100, ng/mL, *n* (%)	14 (32.6)	20 (30.3)	.804	12 (32.4)	12 (32.4)	1.000
Total bilirubin level, μmol/L, median (range)	14.5 (5.9–28.7)	14.1 (6.1–30.3)	.552	15.6 (5.9–28.7)	12.9 (7–30.3)	.611
ALT level, IU/L, median (range)	30 (9–79)	28 (6–152)	.125	30 (9–79)	30 (8–71)	.634
AST level (IU/L), median (range)	28 (16–70)	28 (14–157)	.322	28 (18–70)	32 (15–88)	.957
Serum albumin (g/L), median (range)	42.6 (31.8–52.1)	43.4 (32.5–51.4)	.579	42.6 (31.8–52.1)	43.6 (38.2–51.4)	.294
Prothrombin time, s, median (range)	11.8 (10.1–17.4)	11.9 (10.3–14.6)	.598	11.7 (10.2–14.2)	12.1 (10.3–14.4)	.176
Platelet count, 10^9^ /L, median (range)	105 (64–310)	119.5 (60–238)	.422	105 (64–218)	108 (60–194)	.970
Child‐Pugh class A, *n* (%)	42 (97.7)	64 (97.0)	1.000	35 (94.6)	35 (94.6)	1.000
ICG‐R15 < 10%, *n* (%)	36 (83.7)	55 (83.3)	.958	32 (86.5)	33 (89.2)	1.000
Patients with two tumors, *n* (%)	22 (51.2)	47 (71.2)	.034	20 (54.1)	26 (70.3)	.150
Largest tumor size, cm, median (range)	2.7 (1.4–3)	2.4 (1.1–3)	.026	2.7 (1.4–3)	2.4 (1.2–3)	.050
Total tumor size, cm, median (range)	4.9 (2.2–8)	4.8 (2.1–7.3)	.665	4.8 (2.5–8)	4.8 (2.1–7.3)	.897
Time to recurrence ≤2 years, *n* (%)	19 (44.2)	32 (48.5)	.660	12 (32.4)	15 (40.5)	.469
Located the different lobe, *n* (%)	28 (65.1)	23 (34.8)	.049	23 (62.2)	16 (43.2)	.103
Time span, *n* (%)						
April 2010–April 2016	13 (30.2)	14 (21.2)	.286	9 (24.3)	8 (21.6)	.782

Abbreviations: AFP, alpha‐fetoprotein; ALT, alanine aminotransferase; AST, aspartate aminotransferase; HBV DNA, hepatitis B virus deoxyribonucleic acid; HBV, hepatitis B virus; HCC, hepatocellular carcinoma; HCV, hepatitis C virus; ICG‐R15, indocyanine green retention rate at 15 min; RH, repeat hepatectomy; SD, standard deviation.

### 3.2 Clinicopathologic and operative data at initial resection

As shown in Table 2, the two groups were similar in the number of cases with AFP ≥400 ng/mL, Child–Pugh class A, BCLC stage, major tumor size, and tumor number at initial hepatectomy. The intraoperative circumstance and postoperative pathological results were comparable between the two groups.

**TABLE 2:** Clinical characteristics of patients with HCC for the initial resection.

Variable	Combination (*n* = 43)	HR alone (*n* = 66)	*p* value
Serum AFP ≥400 ng/ml, *n* (%)	9 (20.9)	14 (21.2)	.972
Major tumor size, cm, median (range)	4.5 (1.6–14)	3.8 (1–12)	.076
Tumor number, solitary, *n* (%)	32 (74.4)	56 (84.8)	.177
Complete tumor capsule present, *n* (%)	21 (48.8)	30 (45.5)	.729
Vascular invasion, *n* (%)			
Micro	24 (55.8)	34 (51.5)	1.000
Macro	3 (7.0)	5 (7.6)	.660
Satellites present, *n* (%)	13 (30.2)	14 (21.2)	.286
Tumor grade, *n* (%)			
G1	2 (4.7)	3 (4.5)	1.000
G2	23 (53.5)	32 (48.5)	.610
G3–G4	18 (41.9)	31 (47.0)	.600
Liver cirrhosis, *n* (%)	21 (48.8)	31 (47.0)	.849
Ishak score, median (range)	5 (2–6)	5 (1–6)	.878
Resected margin positivity, *n* (%)	1 (2.3)	0 (0)	.394
BCLC stage, *n* (%)			
0‐A	31 (72.1)	55 (83.3)	.160
B	9 (20.9)	6 (9.1)	.079
Estimated blood loss, mL, median (range)	305 (35–2100)	260 (30–1980)	.194
Intraoperative blood transfusion, *n* (%)	6 (14.0)	4 (6.1)	.163
Extent of liver resection (Major), *n* (%)	10 (23.3)	10 (15.2)	.285

Abbreviations: AFP alpha‐fetoprotein; HCC, hepatocellular carcinoma; HR, hepatic resection; RFA, radiofrequency ablation.

### 3.3 Operative and postoperative data of second surgery

As shown in Table 3, patients in RH group had a longer operation time than patients in combination group (before PSM: *p* < .001; after PSM: *p* < .001). The RH group had more patients with intraoperative blood loss of 501–1000 mL than the combination group (*p* = .034), and the combination group had more patients with intraoperative blood loss of <200 mL than the RH group (*p* = .042). These differences were disappeared after PSM.

**TABLE 3:** Operative and postoperative data of HCC patients with recurrent and multiple tumors meeting the Milan criteria at the time of recurrence.

Variable	Before propensity matching			After propensity matching		
	Combination (*n* = 43)	HR alone (*n* = 66)	*p* value	Combination (*n* = 37)	HR alone (*n* = 37)	*p* value
Type of excision						
All tumors en bloc resection, *n* (%)	—	34 (51.5)	—	—	22 (59.5)	—
Local resection	38 (88.4)	51 (77.3)	.391	32 (86.5)	26 (67.6)	.090
Hemi‐hepatectomy	4 (9.3)	10 (15.2)	.372	2 (5.4)	7 (21.6)	.152
Extended resection	1 (2.3)	5 (7.6)	.400	1 (2.7)	4 (10.8)	.358
Operation time, h, mean ± SD (range)	4.4 (3.7–6.2)	4.9 (4.4–6.8)	<.001	4.3 (3.7–6.2)	5.0 (4.6–6.8)	<.001
Estimated blood loss (mL)						
≤ 200	9 (20.9)	5 (7.6)	.042	6 (16.2)	3 (8.1)	.479
201–500	27 (62.8)	32 (48.5)	.143	25 (67.6)	18 (48.6)	.099
501–1000	7 (16.3)	23 (34.8)	.034	6 (16.2)	13 (35.1)	.062
≥1000	0 (0)	6 (9.1)	.079	0 (0)	3 (8.1)	.240
Adhesions present, *n* (%)	29 (67.4)	39 (59.1)	.379	24 (64.9)	22 (59.5)	.632
Intraoperative blood transfusion, *n* (%)	5 (11.6)	11 (16.7)	.468	3 (8.1)	5 (13.5)	.711
New tumor found by intraoperative ultrasound, *n* (%)	6 (5.6)	11 (7.3)	.703	5 (13.5)	7 (18.9)	.528
Data of resected tumor						
Resected tumor number, *n* (%)	58 (54.2)	151 (100)	—	51 (56.0)	85 (100)	—
Resected tumor size (cm)	2.5 (0.5–3)	2 (0.5–3)	.022	2.6 (0.5–3)	2.2 (0.5–3)	.113
Surgical margin for resected tumor (cm)	2 (1.5–2.5)	1.75 (1–2.5)	.085	2 (1.5–2.5)	1.75 (1–2.5)	.213
Resected margin positivity, *n* (%)	0 (0)	1 (0.6)	1.000	0 (0)	0 (0)	1.000
Microvascular invasion	17 (29.3)	27 (17.9)	.070	13 (35.1)	12 (32.4)	.806
Liver cirrhosis	30 (69.8)	37 (56.1)	.151	27 (73.0)	21 (56.8)	.144
Ishak score, median (range)	6 (2–6)	6 (2–6)	.155	6 (2–6)	6 (3–6)	.243
Tumor grade of resected tumor, *n* (%)						
G1	4 (6.9)	8 (5.3)	.656	3 (5.9)	4 (4.7)	1.000
G2	38 (65.6)	103 (68.2)	.710	33 (64.7)	60 (70.6)	.475
G3–G4	16 (27.6)	40 (26.5)	.873	15 (29.4)	21 (24.7)	.547
MO pathologically, *n* (%)	14 (32.6)	28 (42.4)	.301	11 (29.7)	14 (37.8)	.461
Data of ablated tumor						
Ablated tumor number, *n* (%)	49 (45.8)	—	—	40 (44.0)	—	—
Ablated tumor size (cm)	1.2 (0.5–3)	—	—	1.3 (0.5–3)	—	—
Local tumor control after RFA, *n* (%)	49 (100)	—	—	40 (100)	—	—
Ablated tumor circumstance, *n* (%)						
Insufficient liver volume	30 (61.2)	—	—	26 (65)	—	—
Anatomical position	11 (22.4)	—	—	9 (22.5)	—	—
Excessive blood loss	2 (4.1)	—	—	1 (2.5)	—	—
Significant adhesion	4 (8.2)	—	—	3 (7.5)	—	—
Other	2 (4.1)	—	—	1 (2.5)	—	—
Complication grade, *n* (%)						
Grade I	9 (20.9)	10 (15.2)	.437	8 (21.6)	7 (18.9)	.772
Grade II	8 (18.6)	7 (10.6)	.236	5 (13.5)	4 (10.8)	.722
Minor complication	17 (39.5)	17 (25.8)	.129	13 (35.1)	11 (29.7)	.619
Grade IIIa	5 (11.6)	15 (22.7)	.256	4 (10.8)	6 (16.2)	.496
Grade IIIb	2 (4.7)	7 (10.6)	.478	1 (2.7)	3 (8.1)	.304
Grade IVa	1 (2.3)	3 (4.5)	1.000	1 (2.7)	3 (8.1)	.304
Grade IVb	1 (2.3)	1 (2.3)	1.000	1 (2.7)	1 (2.7)	1.00
Grade V	0	0	—	0	0	—
Major complication	9 (20.9)	26 (39.4)	.044	7 (18.9)	13 (35.1)	.116
Duration of postoperative hospital stay, day	6 (5–14)	7 (5–18)	.115	6 (5–14)	7 (5–17)	.078
30‐day mortality	0	0	—	0	0	—

Abbreviations: HCC, hepatocellular carcinoma; HR, hepatic resection; IM, intrahepatic metastasis; MO, multicentric occurrence.

For resected tumors, smaller tumors were present in RH group than in combination group (*p* = .022) before PSM. There were also no significant differences in surgical margin, Ishak score, microvascular invasion present, liver cirrhosis present and tumor grade of Edmondson and Steiner between the two groups. For ablated tumors, a total of 49 tumors were ablated in combination group. Nine tumors (16.3%) were switched from RH to RFA due to intraoperative accidents. All tumors undergoing RFA were locally controlled under the certification of CEUS or CT.

Treatment‐related complications were summarized in Table 3. Complications were reported according to the Clavien–Dindo grade.^28^ Minor complications, classified as Clavien–Dindo grade I or II, were developed in most patients and were similar between the two groups before and after PSM. Major complications were classified as grade III or higher. There were no significant differences among the subgrades of major complications between the two groups before and after PSM. Nevertheless, the RH group had a higher incidence of major complications than the combination group in general (*p* = .044). After PSM, the difference disappeared between the two groups.

### 3.4 Long‐term survival analysis

**FIGURE 2:**
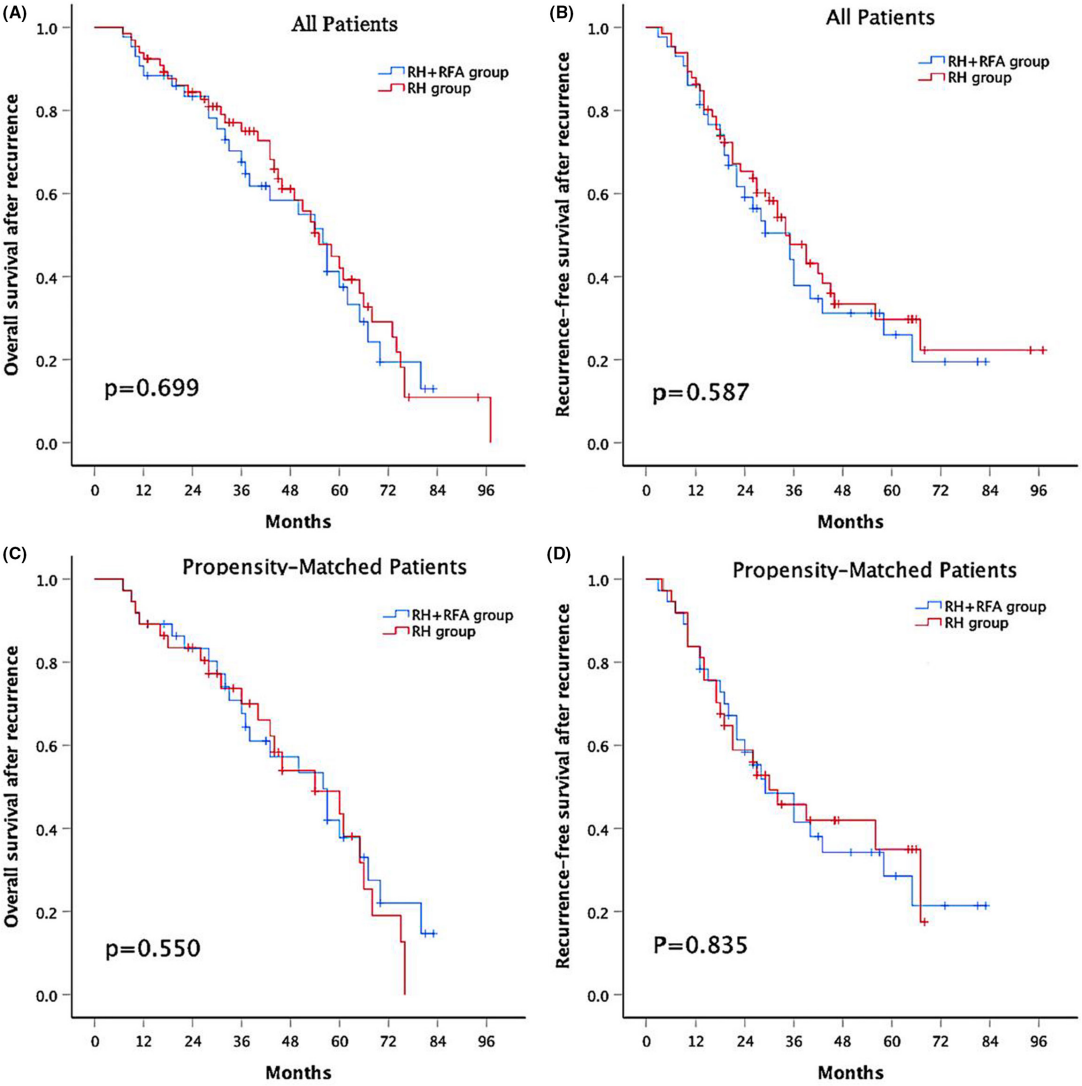
(A) Overall survival after recurrence and (B) recurrence‐free survival rates after recurrence for all study patients who underwent RH combined with RFA or RH alone. (C) Overall survival after recurrence and (D) recurrence‐free survival rates after recurrence for propensity‐matched patients who underwent RH combined with RFA or RH alone. RH, repeat hepatectomy, RFA, radiofrequency ablation.

During a median follow‐up period of 38 months (range 7–97) for all objects, 27 (62.8%) patients in the combination group and 38 (57.6%) patients in the RH group died, and 28 (65.1%) patients in the combination group and 39 (59.1%) patients in the RH group experienced recurrence. The estimated rOS at 1‐, 3‐, and 5‐year was 88.4%, 67.6% and 37.5%, respectively, for the patients in the combination group and 92.4%, 75% and 42.1% for the patients in the RH group (Figure 2A). The cumulative rRFS at 1‐, 3‐, and 5‐year was 86%, 44.2%, and 26%, respectively, for the patients in the combination group and 86.4%, 47.7%, and 29.7% for the patients in the RH group (Figure 2B). Neither the rOS nor rRFS was significantly different between the two groups (*p* = .699 and *p* = .587, respectively).

After PSM, the estimated rOS at 1‐, 3‐, and 5‐year was 89.2%, 67.6%, and 37.8%, respectively for matched patients in combination group and 89.2%, 70%, and 43.5% for matched patients in RH group (Figure 2C). The cumulative rRFS at 1‐, 3‐, and 5‐year was 83.8%, 48.4%, and 28.5%, respectively for matched patients in combination group and 83.8%, 45.8%, and 35% for matched patients in RH group (Figure 2D). The rOS and rRFS were still not significantly different between the two groups (*p* = .550 and *p* = .835, respectively).

### 3.5 Subgroup analysis according to tumor number

**FIGURE 3:**
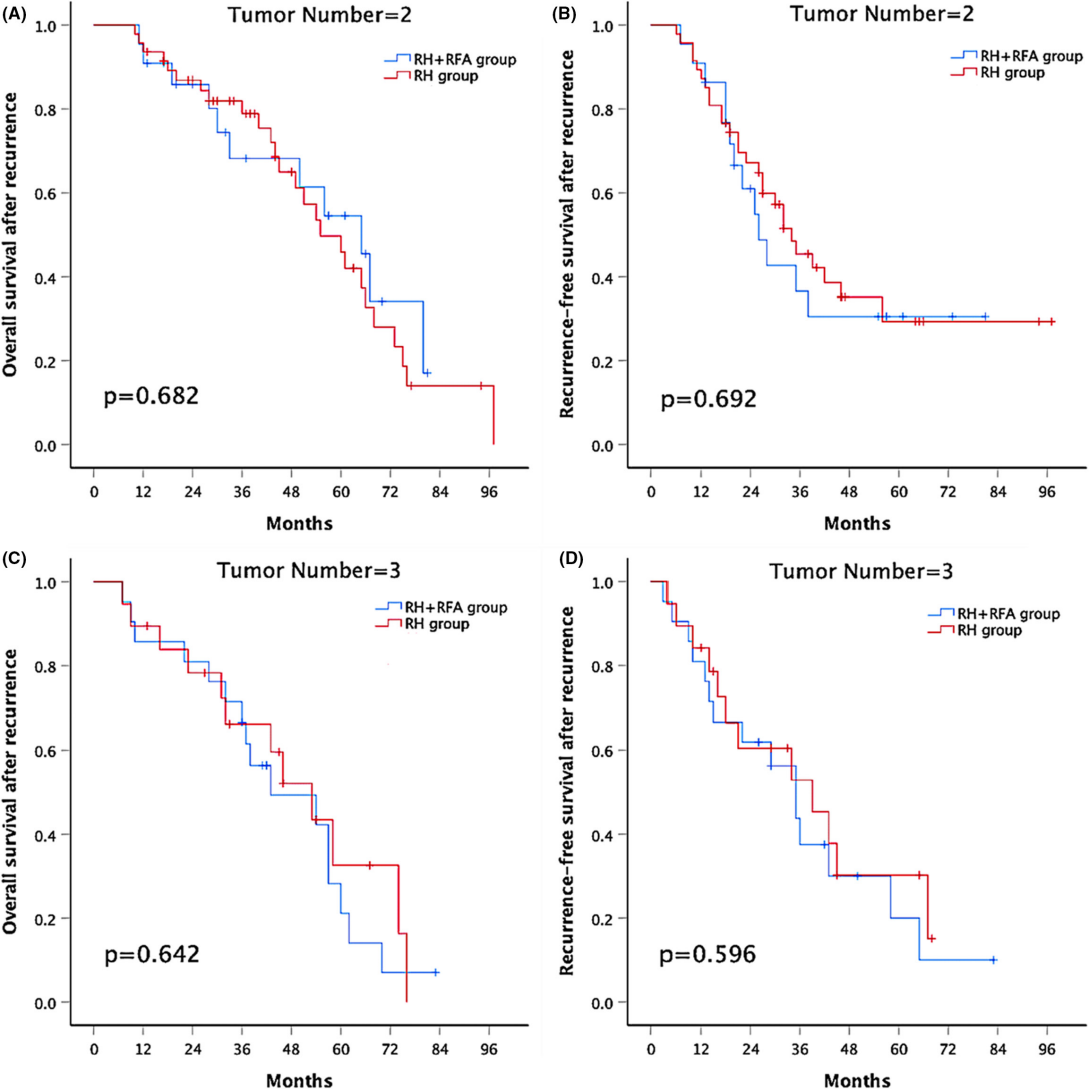
(A) Overall survival after recurrence and (B) recurrence‐free survival after recurrence of patients with two tumors who underwent RH combined with RFA or RH alone. (C) Overall survival after recurrence and (D) recurrence‐free survival after recurrence of patients with three tumors who underwent RH combined with RFA or RH alone. RH, repeat hepatectomy; RFA, radiofrequency ablation.

In the subgroup analyses, the 1‐, 3‐, and 5‐year rOS rates were similar between the combination group and the RH group in patients with two recurrent tumors (90.9%, 68.2%, and 54.6% versus 93.6%, 78.9%, and 49.9%, respectively; *p* = .682; Figure 3A). The 1‐, 3‐, and 5‐year rRFS was also similar between the combination group and the RH group in patients with two recurrent tumors (90.9%, 36.6%, and 30.5% versus 87.2%, 45.4%, and 29.3%, respectively; *p* = .692; Figure 3B). Similar results emerged in patients with three recurrent tumors; the 1‐, 3‐, and 5‐year rOS rates were 85.7%, 66.7%, and 21.2% and 89.5%, 66.2%, and 32.6% in the combination group and the RH group, respectively; *p* = .642 (Figure 3C). The 1‐, 3‐, and 5‐year rRFS rates were 81%, 37.5%, and 20% and 84.2%, 52.9%, and 30.2% in the combination group and the RH group, respectively; *p* = .596 (Figure 3D).

### 3.6 Subgroup analysis according to tumors' anatomic locations.

**FIGURE 4:**
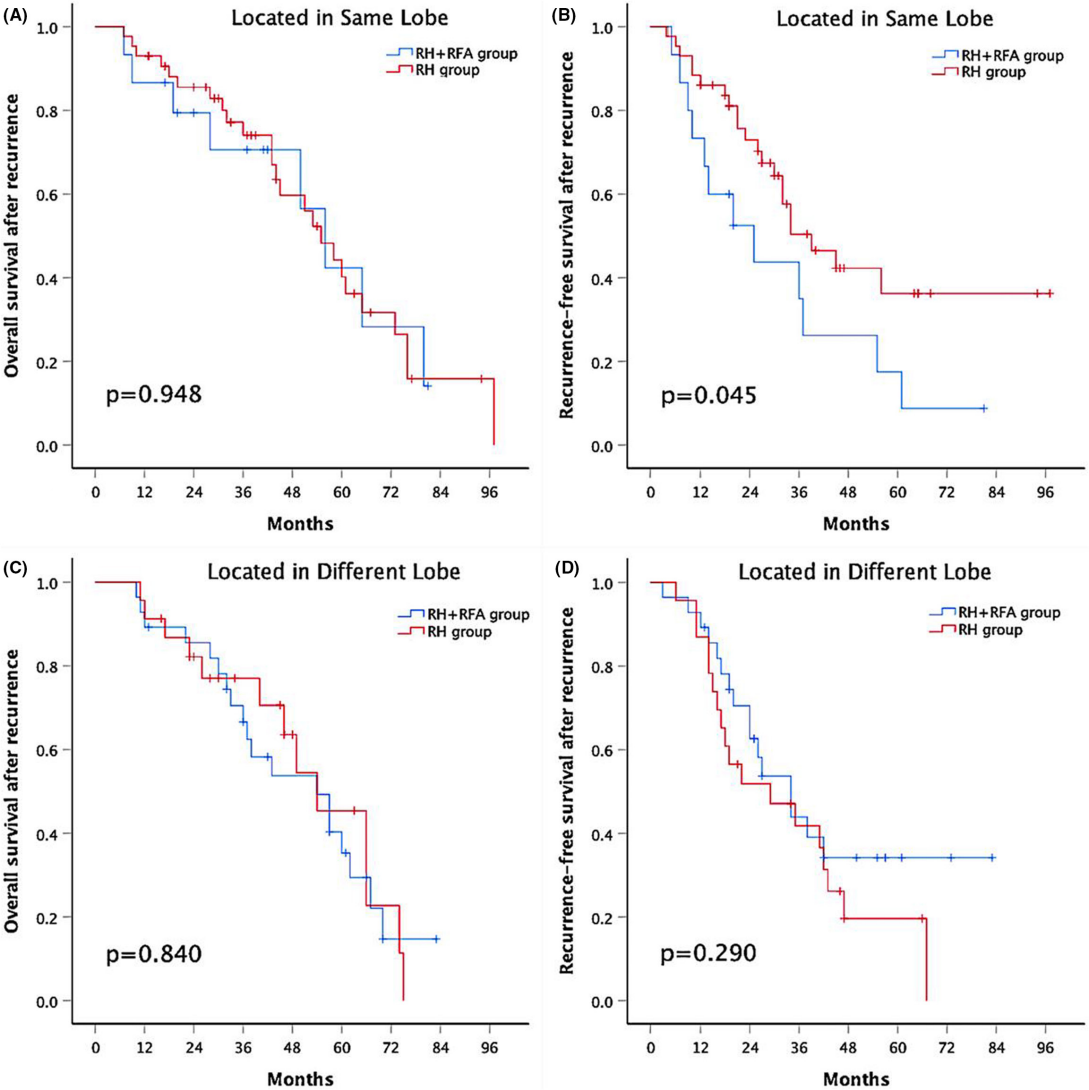
(A) Overall survival after recurrence and (B) recurrence‐free survival after recurrence of patients with tumors located in same lobe who underwent RH combined with RFA or RH alone. (C) Overall survival after recurrence and (D) recurrence‐free survival after recurrence of patients with tumors located in different lobe who underwent RH combined with RFA or RH alone. RH, repeat hepatectomy, RFA, radiofrequency ablation.

In the other subgroup analyses, the 1‐, 3‐, and 5‐year rOS rates were similar between the combination group and the RH group in patients with tumors located in the same lobe (86.7%, 70.6%, and 42.4% versus 93%, 74.1%, and 40.2%, respectively; *p* = .948) (Figure 4A). However, patients with all lesions in the same lobe who underwent RH had a significantly higher rRFS rate than those who underwent RH + RFA (1‐, 3‐, and 5‐year rRFS rates of 86%, 50.4% and 36.2% versus 73.3%, 35% and 17.5%, respectively; *p* = .045) (Figure 4B). Meanwhile, for patients with tumors located in different lobes, there was no significant difference in the 1‐, 3‐, and 5‐year OS rates between the two groups (89.3%, 66.6%, and 35.3% in the combination group vs. 91.3%, 77%, and 45.4% in the RH group, respectively; *p* = .840) (Figure 4C). Similarly, the 1‐, 3‐, and 5‐year rRFS rates were also similar between the two groups (89.3%, 43.9%, 34.2% and 87%, 41.9%, 19.6%, respectively; *p* = .290) (Figure 4D).

### 3.7 Risk‐factor analysis for survival outcome

In univariate analysis, we found that three variables, albumin at recurrence ≤3.5 mg/dL, time to recurrence ≤2 years and IM pathologically, were poor prognostic factors for survival before and after PSM. Variables with *p* < .1 in univariate analysis and operative method were included in multivariate analysis, and we found that time to recurrence ≤2 years and IM pathologically were independent risk factors before and after PSM (Table 4).

**TABLE 4:** Univariate and multivariate analysis of prognostic factors for survival.

	Before propensity matching			After propensity matching		
	HR	95% CI	*p* value	HR	95% CI	*p* value
Univariate analysis						
Sex (M)	1.015	0.629–1.854	.397	1.213	0.710–1.689	.764
Age (≥60 years)	0.875	0.604–1.328	.206	0.945	0.453–1.563	.535
HBsAg (+)	1.131	0.499–2.564	.768	1.341	0.782–1.993	.883
Tumor number at initial resection (*n* ≥ 2)	0.987	0.526–2.505	.487	1.110	0.498–1.536	.268
BCLC stage at initial resection (stage ≥B)	1.453	0.159–4.543	.119	1.421	0.453–3.879	.212
MVI present at initial resection	1.234	0.913–1.924	.300	1.118	0.793–2.034	.542
HBV DNA at recurrence (≥1000 IU/mL)	1.178	0.753–2.077	.325	1.335	0.634–2.314	.237
AFP at recurrence (≥100 ng/mL)	1.216	0.832–1.821	.289	1.256	0.689–1.775	.456
Albumin at recurrence (≤3.5 mg/dL)	1.827	1.046–2.354	.010	1.945	1.210–3.021	.023
Time to recurrence (≤2 years)	1.931	1.141–2.214	<.001	2.467	1.233–3.648	<.001
IM pathologically	2.137	1.178–3.874	.002	2.373	1.034–4.569	<.001
Number of tumors (3)	1.356	0.959–1.757	.140	1.328	0.836–2.540	.221
Site of recurrence (different lobe)	1.582	0.991–2.730	.085	1.684	0.913–3.235	.078
Operative method (RH + RFA)	1.067	0.730–1.726	.752	1.095	0.673–1.987	.783
Time span: April 2010–April 2016	1.322	0.854–2.013	.133	1.189	0.798–2.857	.320
Multivariate analysis						
Albumin at recurrence (≤3.5 mg/dL)	1.434	0.834–2.118	.098	1.459	0.802–2.349	.139
Time to recurrence (≤2 years)	2.234	1.328–4.213	<.001	2.678	1.122–5.034	<.001
IM pathologically	1.985	1.023–4.928	<.001	2.523	1.168–4.760	<.001
Site of recurrence (different lobe)	1.407	0.986–2.324	.112	1.345	0.812–1.983	.220
Operative method (RH + RFA)	1.103	0.680–1.873	.576	0.987	0.770–1.523	.874

Abbreviations: AFP, alpha‐fetoprotein; BCLC, Barcelona Clinic Liver cancer; CI, confidence interval; HBsAg, hepatitis B surface antigen; HBV DNA, hepatitis B virus deoxyribonucleic acid; HR, hazard ratio; IM, intrahepatic metastasis; M, male; MVI, microvascular invasion; *N*, number; RFA, radiofrequency ablation; RH, repeat hepatectomy.

## 4 DISCUSSION

In the future, the treatment of HCC will tend to rHCC due to the characteristic of high recurrence rate. Under scheduled supervision after initial hepatectomy and advancement in imaging technology. Most rHCC are found in the early periods, making surgical treatments beneficial in these patients,^22^, ^29^ and multifocal rHCC at the second resection was not a risk factor.^30^ Although the overall survival outcome of rHCC after surgical intervention have proved tremendously recently, the optimal treatment for early‐stage and multifocal intrahepatic rHCC is still not unifying.

Current BCLC guidelines restrict the indications for surgical resection to patients with multiple tumors because multifocality is associated with poor survival outcome following resection. Therefore, RFA has been recommended for curative treatment of patients with multiple HCCs with Milan criteria except liver transplantation.^31^ Besides, RFA is more technically feasible for multiple HCCs patient with liver cirrhosis or multi‐lobar location. Therefore, RFA has an important role in these patients with limited accessibility of resection for multiple HCCs.^32^ However, some studies have compared the outcomes between RFA and surgical resection for multiple HCCs meeting the Milan criteria. They deemed that resection can provide better RFS compared to RFA for patients meeting the Milan criteria with multiple HCCs.^32^, ^33^ This brings us a new reflection of treatment strategies for recurrent multiple HCCs meeting the BCLC stage A.

Not only that, RH and RFA were also effective treatments for rHCC, and 5‐year survival after RH was from 31% to 56%^30^, ^34^, ^35^, ^36^, ^37^ and after RFA was from 37% to 45.4%.^10^, ^18^, ^37^ However, RH was limited in patients with poor liver function, severe abdominal adhesion, and insufficient residual liver volume. RFA was not applicable for tumors that are on the liver surface or near large vessels or critical organs. Therefore, it would be better to recognize RH and RFA as complementary treatment modalities rather than totally competitive modalities. In this regard, to overcome the limitations of each when used alone, we compared the long‐term outcome of patients with multifocal rHCC meeting the BCLC stage A with RH and RH combined with intraoperative RFA.

RH is more complicated and challenging than initial resection because of the progression of hepatitis, the presence of adhesions and anatomical modifications by previous operation.^38^, ^39^ Abdominal adhesion was present in 29 of the 43 patients in the combination group and 39 of the 66 patients in the RH group. Neovascular structures and ambiguous anatomy resulted in an unplanned increase in intraoperative blood transfusion, operation time, risk of bowl injury, and perioperative complications. As shown in Table 3, shorter operation time, less intraoperative blood loss, and lower rate of major complications was observed in the combination group than in the RH group. Major complications mainly included postoperative bleeding, liver dysfunction and bile leak, which mainly arose in the RH group, probably due to limitations of RH in the treatment of tumors with special locations.^40^ For example, a tumor located near critical bile duct received RH, which frequently brought about large area of parenchymal transection, subsequently prolonged operation time, increased blood loss, and inherently increased incidence of major complications. Our results are consistent with findings of recently published studies of RFA with less trauma in treating rHCC.^22^, ^25^, ^41^

The combination group and RH group have similar rOS and rRFS rates in patients with two recurrent tumors (Figure 3A,B), which is different from our previous results of initial resection for early‐middle‐stage multifocal HCC.^42^ One reason is that we narrowed the tumor criteria from the University of California San Francisco (UCSF) criteria to the BCLC stage A. Because tumor size is an important risk factor for survival outcome of patients with HCC. The other reason may be that the extent of resection of the recurrent tumor is smaller than that of the initial resection when considering the residual liver volume. Moreover, for patients with three recurrent tumors, survival outcomes are comparable between the two groups (Figure 3C,D). The potential explanation is that the number of recurrent tumors played a dominant role in effecting patients' prognosis. Further subgroup analysis of the anatomic location of tumors was performed according to Couinaud's segmentation. RH provided better rRFS than RH combined with RFA for patients with all lesions in the same lobe (Figure 4B), but in the subgroup of tumors located in different lobes, the difference of rRFS was eliminated between the two groups (Figure 4D). There are several reasons that could account for this phenomenon. Firstly, 30 patients (88.2%) in the RH subgroup of tumors located in the same lobe underwent en bloc resection, which removed more nonneoplastic liver parenchyma than the combination subgroup. Second, it was possibly attributable to frequent presence of microlesions that would likely have been eliminated following an en bloc resection in RH group, because some microlesions may grow slowly, especially in less biologically aggressive tumors.^43^ In other words, recurrent tumors may not be completely controlled by RFA. Third, if tumors were located in different lobes, theoretically, there would be more intrahepatic metastases in the wider liver region, thus causing poor outcomes in either rOS or rRFS in the subgroup of tumors located in the different lobes.

Time to recurrence ≤2, also called ER, and IM pathologically were identified as two risk factors in multivariate analysis. Many studies have clearly demonstrated that patients with ER had significant worse survival than those with LR after the second surgery.^12^, ^18^ IM is associated with more aggressive tumor behavior, whereas LR is more likely to be associated with MO with a beneficial survival outcome. The diagnosis of IM or MO is mainly based on histopathological findings reported by the Liver Cancer Study Group of Japan. Although this means is relatively subjective, it is still the most convenient and effective clinical identification method. In our study, 29 patients (67.4%) were defined as IM in combination group, and 38 patients (57.6%) were defined as IM in RH group. The difference in tumor biological behavior between IM and MO leads to the different prognosis.^12^, ^15^ We can also explain that the IM cases might be a consequence of the so‐called recurrent multifocal HCC from clinically latent multiple intrahepatic metastases, which might have already been present at the time of initial surgery. In other words, these cases might be “latent Stage IV” at the time of initial surgery, mistaken for recurrence at the next follow‐up.

The major limitations were the following. Firstly, this is a retrospective study with a small sample size, inherent selection bias was unavoidable, even though propensity score matching, and multivariable regression analysis were used. It needed to be verified by more multi‐center and prospective studies. Second, the outcomes of both RH and RFA partly depend on the expertise and experience of the operators, and we failed to consider the effects of different antiviral drugs that were associated with tumor recurrence during the follow‐up periods. It was difficult to balance and adjust them between the two groups in this retrospective study. Finally, the study spanned a period of more than 10 years, during which HCC treatment has changed significantly. Despite its limitations, our study does provide some valuable references that can aid clinicians in confronting patients with early‐stage multifocal rHCC.

In conclusion, this study showed that tumor which is difficult to be surgically resected could be treated by RFA in order to avoid more blood loss, and RH combined with RFA had a relatively shorter operative time and fewer postoperative major complications. However, in light of tumors' anatomic locations, RH alone may be more suitable for patients whose tumors are located in the same lobe because of better rRFS. How to treat these patients with an optimal mean still worth looking forward to.

## AUTHOR CONTRIBUTIONS

**Yang Huang:** Formal analysis (equal); methodology (equal); writing – original draft (equal). **Liangliang Xu:** Validation (equal); visualization (equal); writing – review and editing (equal). **Min Huang:** Conceptualization (equal); resources (equal); validation (equal). **Li Jiang:** Data curation (equal); resources (equal); supervision (equal); validation (equal). **Mingqing Xu:** Supervision (equal); validation (equal).

## FUNDING INFORMATION

This work was supported by grants from the National Natural Science Foundation of China (81400636), Sichuan Province Key Research and Development Project (2019YFS0203), Key Clinical Research Incubation Project of West China Hospital of Sichuan University (2020HXFH028), and the Key R&D Support Plan of Chengdu Science and Technology Bureau (2021‐YF05‐00703‐SN).

## CONFLICT OF INTEREST STATEMENT

The authors have no relevant financial or non‐financial interests to disclose.

## Data Availability

The datasets generated during and/or analyzed during the current study are available from the corresponding author on reasonable request.
